# The Potential of Stimuli-Responsive Nanogels in Drug and Active Molecule Delivery for Targeted Therapy

**DOI:** 10.3390/gels3020016

**Published:** 2017-05-08

**Authors:** Marta Vicario-de-la-Torre, Jacqueline Forcada

**Affiliations:** 1 Nutra Essential OTC, Alcobendas, Madrid 28108, Spain; martavicario@nutraessential.com; 2 Bionanoparticles Group, Facultad de Ciencias Químicas, University of the Basque Country UPV/EHU, Donostia-San Sebastián 20018, Spain

**Keywords:** stimuli-responsive nanoparticles, nanogels, drug delivery systems

## Abstract

Nanogels (NGs) are currently under extensive investigation due to their unique properties, such as small particle size, high encapsulation efficiency and protection of active agents from degradation, which make them ideal candidates as drug delivery systems (DDS). Stimuli-responsive NGs are cross-linked nanoparticles (NPs), composed of polymers, natural, synthetic, or a combination thereof that can swell by absorption (uptake) of large amounts of solvent, but not dissolve due to the constituent structure of the polymeric network. NGs can undergo change from a polymeric solution (swell form) to a hard particle (collapsed form) in response to (i) physical stimuli such as temperature, ionic strength, magnetic or electric fields; (ii) chemical stimuli such as pH, ions, specific molecules or (iii) biochemical stimuli such as enzymatic substrates or affinity ligands. The interest in NGs comes from their multi-stimuli nature involving reversible phase transitions in response to changes in the external media in a faster way than macroscopic gels or hydrogels due to their nanometric size. NGs have a porous structure able to encapsulate small molecules such as drugs and genes, then releasing them by changing their volume when external stimuli are applied.

## 1. Introduction

The introduction of therapeutic agents into the body using different administration routes must overcome different and efficient immunological barriers. After administration, therapeutic effects of the drugs are limited or reduced due to the bioavailability or partial degradation before reaching the target site. Several therapeutic approaches (mainly nanosystems) have been developed to overcome the biological barriers, increase drug bioavailability, and achieve a major effect in the desired target. As many therapeutic effects of the drugs are limited or reduced due to the partial degradation that occurs before they reach the desired target, the need of a suitable carrier is envisaged as a solution. For the administration of therapeutics, the use of stimuli-responsive systems at the nanoscale (nanocarriers) able to overcome and bypass the different biological barriers is considered an efficient way to achieve this objective.

In recent years, great interest has been directed to environmentally responsive polymers capable of undergoing structural changes in response to an external stimulus due to the possibility, by using these polymers, of forming responsive polymeric nanoparticles, such as nanogels (NGs). Stimuli-responsive nanogels are cross-linked colloidal particles, which can swell by absorption of large amounts of solvent, but do not dissolve due to the constituting structure of the polymer network, physically or chemically cross-linked. As other nanocarriers, such as liposomes, solid nanoparticles, and dendrimers, NGs have a size comprised in the colloidal scale, ranging from 1 to 1000 nm. Nanoparticles (NPs) are solid and spherical structures, submicron-sized, prepared from polymeric materials and/or metallic compounds. NPs can be classified as nanocapsules (the active agent forms a matrix combined with the polymer-forming the NP) and nanospheres (the active agent constitutes the core surrounded by polymeric material). On the other hand, NGs are considered as soft NPs mainly due to their high water content that can fluctuate, modifying, in turn, the NG structure. In this sense, NGs can swell by absorption (uptake) of large amounts of solvent (commonly, water) but they do not dissolve due to the constituent structure of the polymeric tridimensional network, physically or chemically cross-linked. The potential biotechnological applications of NGs are based in their multi-stimuli responsive nature that allows NGs to change their inner structure reversibly. NGs respond to external changes by suffering phase transitions in a faster fashion than macroscopic gels due to their nanosized scale, and thus NGs achieve an efficient therapeutic delivery at the pathological areas. The most common reversible phase transition behavior that NGs show is the ability to change their volume that goes from a polymeric solution (swollen form) to a hard particle (collapsed form). Swelling and shrinking is caused by conformational changes of the subchains between two nearby cross-linking points inside the gel network. This behavior is governed by the balance and result of the competition between repulsive intermolecular forces acting to expand the tri-dimensional polymer network of polymeric chains loosely cross-linked forming the nanogel and attractive forces that act to shrink it: swelling occurs when the ionic repulsion and the osmotic forces are higher than the attractive forces, i.e., hydrogen bonds, van der Waals and hydrophobic interactions. NGs are environmentally sensitive to (i) physical stimuli, such as temperature, ionic strength, magnetic or electric fields; (ii) chemical stimuli (pH, ions presence, specific molecules) and (iii) biotechnological stimuli such as enzymatic substrates, affinity ligands, cell receptors, etc.) [1].

NGs are considered versatile carriers for biomedical applications, especially for cancer therapy because these multi-stimuli soft nanoparticles take advantage of the abnormalities present in tumors such as a lower pH, an increased reductive environment and a higher temperature. In addition, drug bioavailability is increased once it is included into the NGs [2]. On the other hand, conventional antitumor pharmaceutical formulations show a low drug bioavailability and a short-half life after administration, subsequently an increased frequency of administration and elevated doses are necessary to achieve a therapeutic effect. Consequently, systemic toxicity and unwanted effects appear in patients. Indeed, the effectiveness of conventional therapies is mainly determined by the appearance of side effects. Therefore, drug delivery systems (DDS) and nanosized carriers in particular, are positioning themselves as valid alternatives to prevalent cancer treatments leading to an enhanced anti-tumor effect, reduced toxicity, and unwanted effects. Although the translation of basic research to clinical assays and finally getting marketed formulations is a complex and time-consuming process, the extensive research work developed over the last decades has reached the marketing of several nano-medicines. As examples, Doxil^®^ and Abraxane^®^, a Doxorubicin (Dox)-loaded PEGylated liposomes (LPs) [3] and Paclitaxel albumin-stabilized NPs [4] respectively, are approved for U.S. Food and Drug Administration (FDA) for cancer treatment.

In this sense, nanosized carriers (20–200 nm) show a superior therapeutic effect due to the passive targeting, a phenomenon that gives the ability of nanocarriers to achieve the tumor site showing an enhanced permeability and retention (EPR) effect. This phenomena is due to (i) the weak lymphatic drainage in the tumor and (ii) the ability of nanocarriers to cross through the gap junctions in the novel endothelium tumor blood vessels (neovascularization or angiogenesis) that are abnormally large (compared to healthy tissues) in solid tumors and inflammatory tissues [5]. Innovative trends in designing nanocarriers are focused on active targeting, where the nanosized carrier is surface-functionalized to recognize specific receptors at the target site. One of the main advantages of NGs is that they can be easily functionalized during synthesis, so both passive and active targeting may enhance drug efficacy. Indeed, NGs, as other nanosized DDS, can also carry inorganic loads, such as Ag, Au, or magnetic NPs that allow the diagnosis and the treatment in a single carrier, thus allowing to fit the therapy according to the patient requirements (personalized medicine) and improving the effectiveness of the therapy that otherwise may not have been as successful [6,7]. Along this line, stimuli-responsive NGs are able to combine in a unique carrier responsiveness to environmental factors, specificity for a particular receptor, a controlled release of the active agent from their polymeric network, cyto- and bio-compatibility, stability, and prolonged blood circulation times [8,9,10,11,12]. In addition, poly(ethylene glycol) (PEG) is usually added to NGs to extend their blood circulation, enhancing stability, preventing protein binding, avoiding mononuclear phagocytic system (MPS) and, protecting otherwise fragile molecules after administration [12].

Although stimuli-responsive NGs are mainly employed for cancer therapy, these versatile carriers are useful for selectively targeting other diseases as is discussed in this review.

Despite the deeply and extensive work to boost NGs to clinical practice, several disadvantages have been necessary to overcome and only a few NGs-based formulations have been tested in clinical trials [13,14,15]. Although, NGs can be easily functionalized to target the site of action and present a stimuli-responsive behavior “in vitro”, the “in vivo” correlation is difficult and needs further optimization. Biodistribution and efficacy of NGs as DDS after administration is determined by parameters such as particle size, stability of the loaded-nanogels, surface charge, and properties from functionalized groups, ability to reach and deliver the cargo at the target site avoiding healthy tissues and biodegradation [16,17,18,19]. Therefore, more studies involving the behavior of the NGs after administration regarding these items are necessary. Moreover, biocompatibility and biodegradability studies are required to determine the biodistribution and elimination of NGs-based formulations after “in vivo” administration. For that reason, the present work attempts to briefly indicate the progress made in synthesis, functionalization, “in vitro” features necessary for the NGs to be employed as DDS, and “in vivo” efficacy studies of multifunctional nanogel formulations.

## 2. Approaches for the Production of Stimuli-Responsive Nanogels

As commented previously, an ideal drug delivery carrier should have a small particle size, biocompatibility, biodegradability, high encapsulation efficiency, site-specific therapeutic delivery and retention at the site of action for a long period of time, prolonged circulation time, and avoidance of nonspecific interactions occurring with the environment (body inner structures). In the case of polymeric nanocarriers, different synthetic processes can be used to produce these particular nanoparticles, among them polymerization in dispersed media of the adequate monomers under specific reaction conditions will give as a result new responsive polymeric nanoparticles with potential applications in the biomedical field as delivery systems for active agents (drugs, molecules, and metallic and magnetic nanoparticles, among others). Among the polymerization methods commonly employed for the synthesis of NGs are emulsion polymerization, precipitation polymerization, inverse microemulsion polymerization, anionic copolymerization, and cross-linking between neighboring chains.

**Figure 1:**
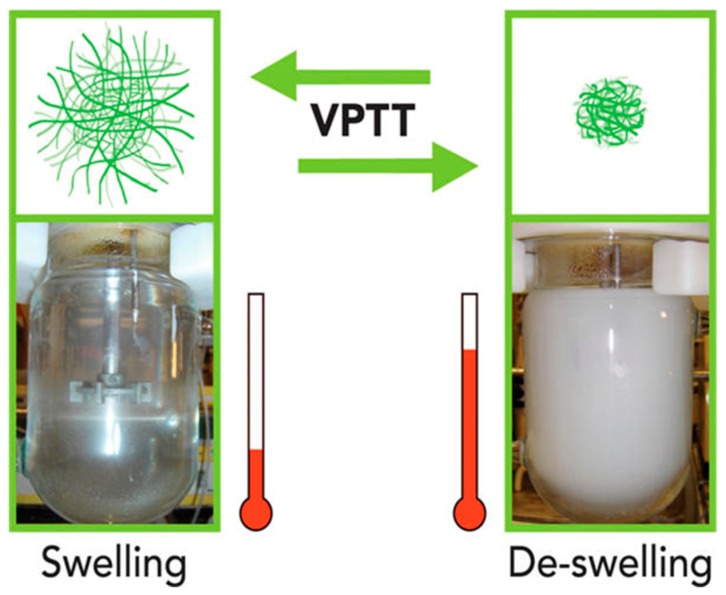
A thermo-responsive nanogel in its swollen state (T < VPTT) and in its collapsed state (T > VTPP) (Reprinted with permission from reference [1]. VPTT = volume phase transition temperature. Copyright 2014 ACS).

NGs can respond to external stimuli, and among them, the most studied one is temperature because changes in temperature are common in pathological states and can be easily applied externally. From a biotechnological point of view, these NGs are very interesting since they can undergo a volumetric phase change releasing the cargo when a temperature change is produced. In this line, the design and controlled production of thermo-responsive nanogels have received considerable interest due to their unique feature to swell at low temperatures and be collapsed at high ones in aqueous solutions, showing a volume phase transition temperature (VPTT) (see Figure 1). The adequate monomer selection and a controlled polymerization process can lead to obtain thermo-responsive and biocompatible NGs with VPTTs close to physiological temperature (in healthy and unhealthy conditions) being very attractive for bio-applications, such as drug delivery.

Among the responsive polymers employed in the synthesis of sensitive nanogels, poly(alkylacrylamides), more specifically poly(*N*-isopropylacrylamide), (PNIPAM) is the thermo-responsive polymer most frequently employed. Although there are studies that indicate that PNIPAM entails a low toxicity grade, PNIPAM is more present in NGs synthesis destined for biomedical application over the past few years [20]. Another thermo-responsive polymer, poly(*N*-vinylcaprolactam) (PVCL), is considered adequate for delivery systems and to be used in biomedical devices, due to its biocompatibility and VPPT in the physiological temperature region (32–38 °C). This allows PVCL to be considered as an adequate material for the design of biomedical devices and to be useful in drug delivery systems [1].

On the other hand, NGs able to respond to changes in pH (pH-sensitive NGs) are also promising. These NGs can swell when the pH approximates the pKa of the ionic monomer incorporated by copolymerization in the cross-linked chains constituting the particles. These nanogels are useful in the case of releasing a biologically active compound in a physiological medium in which the main characteristic is the change in pH. pH-sensitive nanogels are composed of cross-linked polyelectrolytes with weakly acidic (i.e., carboxylic) and/or weakly basic (i.e., amino) group(s) which can be used either as proton donors or receptors, or through a combination of both. The choice of polymer depends on the physiological conditions of the target in which the delivery is needed. pH-responsive nanogels are able to swell in response to small pH variations, showing a volume phase transition pH (VPTpH). Below this transition pH, nanogel particles are swollen and above it, are collapsed. The volume change is ascribed to the enhanced electrostatic repulsion among charges within the polymer network that appears due to the ionization of ionizable groups varying the pH. Among the polymers employed in the synthesis of pH-responsive NGs are poly-(acrylic) acids (PAA), methacrylic acid (MA), polyethylenimine (PEI), poly(2-*N*,*N*-(diethylamino)ethyl methacrylate) (PDEAEMA) derivatives, etc. The biomedical application of these NGs is focused on pH changes, a phenomenon that occurs regularly in the body at both healthy and unhealthy conditions. The selection of the polymer depends on the pH of the target site, i.e., gastrointestinal tract (with a pH gradient), inflamed tissues (a more acidic pH), cancer cells and endolysosomes (acidic pH in comparison with healthy cells and cytoplasm, respectively) [21,22].

Copolymerization leads to obtaining multi-sensitive NGs by incorporating polymers able to respond to different stimuli. This is the case of pH- and temperature-responsive NGs obtained by emulsion copolymerization of VCL with an ionizable comonomer (acrylic acid) as in the case of the preparation of acid containing PVCL-based nanogels [23,24]. More recently, the synthesis of poly(2-(diethylamino)ethyl) methacrylate (PDEAEMA)-based nanogels with dual pH and temperature sensitivities [25], as well as the synthesis of PDEAEMA/PVCL-based core-shell thermo- and pH-responsive nanogels prepared by seeded batch emulsion polymerization [26], have been reported.

Apart from the different polymerization techniques used, there are other options for the production of nanogels. Among them, autoclaving is an alternative approach for nanogels synthesis when components that comprise the system withstand high temperatures while preserving their functionality. Montanari et al. [27] suggested an innovative approach for obtaining sterilized nanogels by using an autoclave. In this work, gellan- and hyaluronic acid-cholesterol derivatives were first synthesized, dispersed in aqueous solutions and then, sterilized by autoclaving to achieve preformed polymeric-based NGs. The temperature (121 °C) and pressure (1.1 bar) for 20 min in an autoclave promoted the interactions between the hydrophobic domains of cholesterol moieties and the hydrophilic chains of the polysaccharides. Particle size was close to 200 nm and 350 nm for gellan- and hyaluronan-cholesterol based nanogels respectively and the mean size remained stable at 4 and 37 °C for 1 week and 1 month correspondingly. Furthermore, levofloxacin was added to the aqueous suspensions to get drug-loaded NGs by autoclaving. There was a slight increase in size of the levofloxacin-loaded NGs compared to blank ones (of about 30 nm) although the encapsulation efficiency (EE) was low, close to 5% in gellan- and hyaluronan-cholesterol-based NGs. One of the most remarking points of this study is that nanogels obtained by autoclaving showed properties such as size and drug capacities comparable to nanohydrogels obtained by the sonication approach.

On the other hand, gamma radiation and e-beam sterilization are the most employed methods for both the “in situ” formation and sterilization of nanogels. The synthetic “in situ” approach is based on irradiation of semi-dilute aqueous solutions of preformed polymers. As a consequence, many radicals are generated simultaneously along each polymer chain leading to intramolecular recombination and the subsequent formation of nanogels [28,29,30]. Besides obtaining sterilized nanosystems, “in situ” radiation methods achieve NGs without involving organic solvents, surfactants, cross-linkers or chemical initiators.

### 2.1. Stimuli-Responsive Nanogels

As a consequence of the colloidal and morphological features of these stimuli-sensitive soft nanoparticles, nanogels can contain active agents and release them by changing their volume when changes in the media (external stimuli) occur. Taking advantage of biological stimuli such as pH and temperature, NGs can release the drug in a controlled fashion at the site of action, thus providing enhanced therapeutic outcomes.

The pH in blood and healthy tissues is close to 7.4 while in tumors or inflammatory tissues it drops 0.5–1 units. Moreover, a pH gradient is observed during cellular uptake, since the carriers first reach the internal cell structures from endosome (pH about 6) to lysosome with a pH of 4.5 [31]. A redox gradient is also present between the oxidizing extracellular environment and the reductive intracellular medium. The most abundant reductive peptide, glutathione (GSH), is found to be 10–100-fold more concentrated in the cytosol (1–10 mM) than in the extracellular fluids (10 µM) [32]. In addition, cancer cells show higher GSH concentrations than healthy ones [33]. Relating to temperature, a local hyperthermia appears in cancer cells and inflammatory diseases. Noteworthy, tumor diseases present several of these altered conditions (pH, temperature, and GSH concentration), thus cancer cells are interesting targets for the development of non-conventional therapies based on stimuli-responsive NGs as drug delivery systems.

#### 2.1.1. Thermo-Responsive Nanogels

Thermo-responsive nanogels undergo a volume change around the volume phase transition temperature (VPTT), which can be tuned by changing their hydrophilic and hydrophobic content [20,34] and, in the case of poly(*N*-vinylcaprolactam) (PVCL)-based NGs, the VPTT can also be modified according to polymer chain length and concentration [35]. Furthermore, PVCL-based NGs have demonstrated their suitability for biomedical applications compared to those based on PNIPAM, which have shown higher toxicity “in vitro” due to the formation of unwanted toxic amide compounds after degradation in acidic conditions [20]. In addition, thermo-responsive PVCL oligomers have antibacterial properties by inhibiting biofilm production and have shown efficacy in reducing virulence of *Escherichia coli* [36].

Thermo-responsive NGs are employed in the treatment of different diseases, cancer being one of the most targeted pathologies. Frequently, an external heating source is applied to enhance the efficiency and selectivity of these nanocarriers. As an example, fragile molecules such as DNA have been condensed with polyethylenimine (PEI) covalently bonded with PNIPAM to improve transfection efficiency by accumulation when local hyperthermia is applied [37]. On the other hand, 5-Fluorouracil (5-FU) was loaded in biodegradable NGs based on chitosan and PVCL, a system that showed non-toxicity and a controlled release above VPTT (38 °C) [38].

#### 2.1.2. pH-Responsive Nanogels

pH-Sensitive NGs are cross-linked nanoparticles with acid or basic groups with a swelling de-swelling behavior depending on pH. NGs with a volume phase transition pH (VPTpH) in the physiologically relevant range are of great interest. Cationic NGs are in a collapsed state at pH values above the pKa, but on decreasing the pH below the pKa, NGs are swollen due to the protonation of the network and the electrostatic repulsion between positively charged groups. According to this behavior, cationic NGs are key carriers, in cancer therapy especially, because unhealthy tissue and cells have lower pH than healthy ones, the swelling of the nanogels at acidic pHs being interesting.

pH-responsive NGs can be produced by assembling natural based-polymers or by polymerization. In the case of polymerizing methacrylic acid (MAA) with PEG diacrylate, nanogel particles with VPTpH close to the pKa of MAA (4.7), have a decrease in the hydrodynamic diameter of 40 nm or approximately 150 nm depending on the surfactant concentration used [39]. NGs based on poly(2-*N*,*N*-(diethylamino)ethyl methacrylate) (PDEAEMA) with VPTpH in the physiological range are very interesting nanoparticles. Indeed, the hydrophobicity of the NGs can be modulated by the degree of protonation of the amine groups in the DEAEMA units and thus, the capture of molecules and its release can be controlled by a variation of the pH of the medium [40]. PEG cross-linked acrylic NGs were synthesized by inverse emulsion polymerization and loaded with curcumin (encapsulation efficiency (EE) close to 70%). According to the cationic nature of the NGs, swelling occurs at a pH above the pKa of the carboxylic groups of the acrylic acid and the release rate is faster at acidic pH [41].

On the other hand, pH-responsive NGs can be synthesized by assembling pH-responsive natural polymers or by functionalizing natural polymers with pH-sensitive molecules. Chitosan, a natural pH-responsive polymer, can enhance its pH-sensitivity by polymerizing with pH-responsive monomers, thus leading to biocompatible and pH-activated NGs for tumor therapy [42,43]. Otherwise, chitin was conjugated with poly(l-lactic acid) (PLA) and loaded with Dox for the treatment of hepatic carcinoma. Chitin-PLA nanogel particles are swollen at a pH below the pKa of the polyelectrolyte (6.1) and the swelling ratio decreases when Dox is conjugated due to a reduction in the number of reactive functional groups in chitin-PLA NGs. Cell internalization of Dox-loaded chitin-PLA nanogels was favored by pH differences in the intracellular media [44]. Combined amphiphilic polypeptide-based block copolymers such as methoxy poly(ethylene glycol)-*b*-poly(l-glutamic acid), loaded with Dox, were developed for lung cancer therapy. The ionization degree of poly-l-glutamic acid determines the pH-dependent responses (ζ potential and Dox release) besides showing high anti-tumor efficacy, low toxicity and favorable hemocompatibility [45].

#### 2.1.3. Light Responsive Nanogels

Light is an attractive resource as an external stimulus to obtain a controlled release of active molecules, since it can be externally applied with high spatial and temporal precision. Furthermore, there are several parameters (wavelength, light intensity, duration of exposure) to control in order to get the desired effect in vivo. Light responsive nanogels are classified as (i) NGs fabricated from light responsive polymers that contain photoactive groups such as azobenzene, spirobenzopyran, triphenylmethane, or cinnamonyl and (ii) a hybrid system composed of NPs containing noble metals such as Au and/or Ag. Light responsive NGs containing light responsive polymers are able to change their size, shape, or ionic nature when irradiation is applied. For example, Patnaik et al. [46] combined azobenzene with dextran moieties to form azodextran-based NGs by a self-assembly physical procedure. On irradiation (trans–cis) isomerization of the hydrophobic azobenzene moiety in the cross-linked side chain of hydrophilic dextran molecules results in weakening of hydrophobic interactions, and in matrix relaxation, that entails size changes in the azodextran-based NGs. When a 365 nm wavelength is irradiated, these NGs undergo photoisomerization that leads to the collapse of NGs and the content’s release. After loading with two model drugs (rhodamine and salicylic acid), photoisomerization is able to control “in vitro” drug release according to the pH and salt concentrations of the media. However, these light responsive NGs are rarely used as drug delivery systems because they need a UV or visible short wavelength light that entails major drawbacks. For example, these kinds of light are strongly absorbed by skin and tissue and therefore cannot be used for deep-tissue triggering as it will damage tissues even at much lower power [47].

On the other hand, the second light responsive NGs category is hybrid NGs containing metallic NPs and a polymer sensitive to temperature changes. In these cases, Ag and Au particles are mainly used, that absorb NIR (near infrared) light (650–900 nm) to generate NIR heat that is minimally absorbed by skin and tissues. In addition, Au NPs are considered the most useful for drug delivery because they have no toxicity and are the most stable metal at the nanoscale [48]. For example, Kawano et al. [49] fabricated a NIR light-responsive drug delivery platform based on Au-Ag-nanorods (Au-Ag NRs) and PNIPAM polymer by a process combining colloid-template polymerization and silica etching. When irradiation is applied by a NIR laser, NIPAM-coated gold nanorods demonstrated photothermal phase transition and accumulation in local targeted sites that were irradiated “in vivo”.

Additionally, more works involving multi responsive NGs are describe throughout the text.

#### 2.1.4. Magnetic Nanogels

Superparamagnetic iron oxide nanoparticles (SPIONs) (≤10 nm in diameter size) have been extensively evaluated as DDS, diagnosis, therapeutic and contrast agents in magnetic resonance imaging (MRI) due to their ability to respond when a magnetic field of moderate intensity is applied. This response disappears when the magnetic stimulus is removed avoiding aggregation and residual magnetization. Coating magnetic NPs with biocompatible compounds (citric acid, dextran, and polymers) improves NPs stability preventing agglomeration [50,51]. Indeed, the incorporation of magnetic properties to NGs provides a magnetic response that creates new opportunities for therapeutic and diagnostic fields. Besides magnetic NPs, nanogels can also incorporate into their structure other inorganic moieties such as Au, Ag, or fluorescent quantum dots (QDs) NPs, forming hybrid NGs that can be used for imaging, diagnostic and therapeutic “in vivo” applications [52,53].

**Figure 2:**
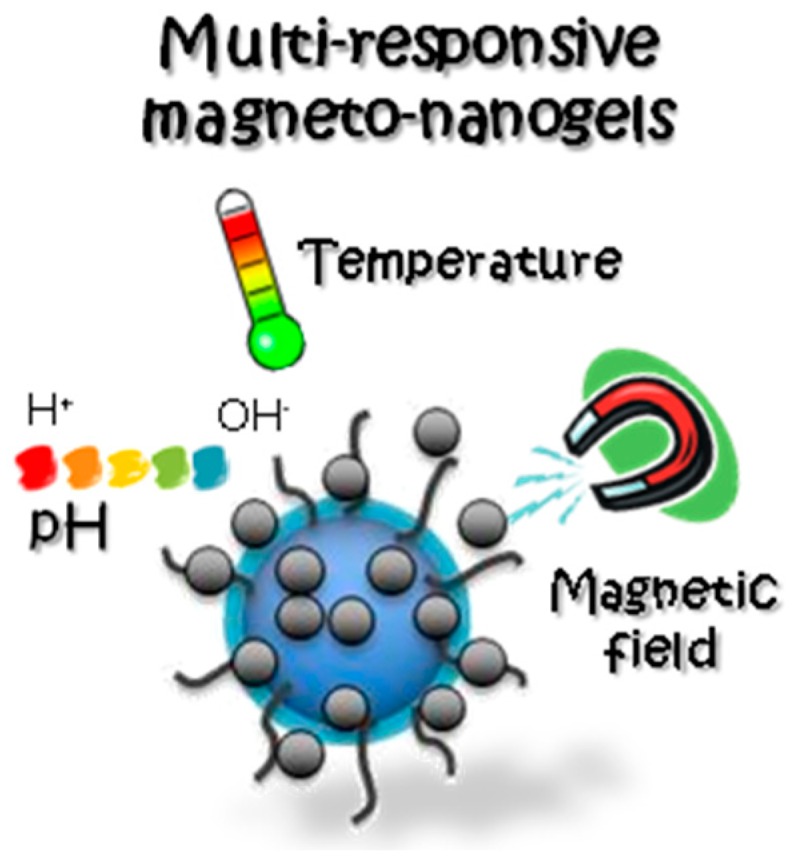
Schematic representation of a multi-stimuli nanogel particle with encapsulated magnetic nanoparticles sensitive to pH, temperature, and magnetic field. (Reprinted with permission from reference [54]. Copyright 2016 Wiley).

The most widespread and innovative approach is to use novel magneto-nanogel formulations for multipurpose applications by combining magnetic NPs with multi-responsive polymer-based NGs (Figure 2) [54].

### 2.2. Targeted Nanogels

Although nanosized carriers enhance effectiveness by EPR effect in tumors and inflammatory diseases, usually active targeting is required to achieve an improved therapeutic efficacy and importantly to reduce drug toxicity. Nanogels can be easily functionalized with specific targeting groups to provide a selective delivery of the active agent in the target cells, organs, or tissues. The targeting groups should (1) provide binding or anchoring of the carriers to the target site and consequently; (2) improve the internalization of the NG and the specific delivery of the cargo and (3) do not cause adverse effects. There are several targeting groups that can be used for an effective targeted therapy such as receptors, ligands, nucleic acids, peptides, and hormones [55,56,57,58,59].

One of the most employed targeting groups is folic acid (FA) which binds selectively to folate receptors (FR), overexpressed in many human tumors [60]. Nevertheless, the addition of FA produces a slight decrease in NGs water solubility due to the incorporation of hydrophobic groups [61], so PEG is usually added as a linker to overcome this issue besides providing an increased drug-loaded NGs bioavailability [62]. Following this line, Nukolova et al. [63] used diblock copolymer poly(ethylene oxide)-b-poly(methacrylic acid) (PEO-b-PMAA) to form NGs conjugated with an optimal number of folate molecules (FA-NGs mean size 110.2 nm). FA-NGs were loaded with cisplatin or Dox with no significant changes in size or stability when the ratio drug/polymer was as much as 1/2 and the relation folate/polymer was 0.1/0.3 µmol folate/mg polymer. After lyophilization and re-dispersion, drug-loaded NG slightly increased their size (152 nm). In any case, both loaded-FA-NGs released the drugs faster at pH 5.5 than at physiological pH due to the protonation of the carboxylic groups from PMAA in acidic media. To determine the effectiveness of the FA group, FA-NGs were incubated with human ovarian cancer cells, A2780, that overexpressed the FR. The uptake of FA-NGs by this cancer cell line exceeded the uptake of untargeted NGs, as expected. Furthermore, FA-NGs selectively recognized FR-positive cells and were successfully internalized or membrane-bonded to these cells. Besides showing no toxicity after IV injection in mice suffering ovarian cancer, cisplatin-loaded FA-NGs inhibited tumor growth, did not produce body weight loss and increased mice lifespan demonstrating a superior anti-tumor activity compared to untargeted NGs.

In recent years, hyaluronic acid (HA) has emerged as a promising candidate for targeting and intracellular delivery of therapeutic agents because HA interacts with cells via CD44 receptor [64], which is present at normal levels in epithelial, hematopoietic and neuronal cells, among others. Moreover, CD44 is significantly overexpressed in carcinomas such as lymphoma, breast, colorectal and lung [65]. Adding a moiety of HA to NGs may increase nanocarrier selectivity and enhance the effect of the drug [66]. Park et al. [67] prepared NGs based on low molecular weight HA chemically modified by acetylation. Authors synthesized three NGs, Ac-HA_LM_-1, Ac-HA_LM_-2, Ac-HA_LM_-3 with different degrees of acetylation: 0.84, 2.09, and 2.86 acetyl groups per 1 unit of HA, respectively. Dox was loaded in these NGs and a higher degree of acetylation improved the EE (59.25%, 83.46% and 93.10% for Ac-HA_LM_-1, Ac-HA_LM_-2, Ac-HA_LM_-3, correspondingly). Similarly, the degree of substitution of HA-NGs resulted in a lower burst effect in the “in vitro” Dox release studies. These results are attributed to the degree of substitution of HA since the presence of acetyl groups makes more hydrophobic cores increasing NGs hydrophobicity and thus, assisting Dox encapsulation and release. The specific interaction between Dox-HA-NGs and tumor cell line HeLa was also monitored. The Dox-loaded HA-NG selectively bound to HeLa cancer cells overexpressing CD44 receptor. The specificity of these systems was clear when Dox-HA-NGs were co-incubated with free HA and the interaction between drug-loaded NGs and cancer cells was reduced due to the inhibition produced by free HA.

Asialoglycoprotein receptor (ASGP-R) is dramatically increased in hepatocellular carcinoma. To target the DDS to tumor hepatocytes surfaces, nanocarriers include carbohydrates able to selectively bind to ASGP-R [68,69]. According to this concept, NG based on PVCL and MAA were conjugated with galactose and synthesized by emulsion polymerization (138.4 nm in the swollen state at 25 °C and 102.4 nm in the collapsed state at 37 °C). NGs’ biodegradability comes from the initiator, *N*,*N*-bis(acryloyl) cystamine, a disulfide-bond containing cross-linker, which after GSH exposure allows the disintegration and shape irregularity of NGs. To achieve a therapeutic anti-cancer effect, multi-responsive NGs were loaded with Dox. Dox release “in vitro” was pH-dependent, thus increasing the release rate when decreasing the pH due to the protonation of both MAA segments and Dox at acidic pH. Additionally, Dox release from NGs was enhanced in an enriched media containing 10 mM GSH, as expected. The efficiency targeting the tumor cells was assessed in HepG2 cells that overexpress ASGP-R, and HeLa cells as negative control. The half maximal inhibitory concentration (IC50) for free Dox and Dox-loaded targeted NGs was 0.7 and 1.08 µM, respectively in HepG2 cells indicating that Dox is efficiently released from NGs in liver cells. Otherwise, the IC50 for free Dox and Dox-targeted NGs was 0.8 and 0.4 µM, correspondingly in HeLa cells suggesting that galactose-functionalized NGs had excellent selectivity for ASPG-R and suitable controlled drug release in tumor hepatic cells [70].

Functionalized NGs can selectively deliver the drug in the target cells/tissues/organs. In that sense, as the knowledge about biomarkers and tumor cell characteristics is rapidly growing nano-medicine is also improving. In fact, NGs can be considered as emerging therapies successfully directed to patient requirements.

### 2.3. Multi-Responsive Nanogels

The multi-modality trend provides a whole approach by combining MRI, visible targeting, targeted thermo- and pH-sensitive chemotherapy and optic sensors, leading to an improvement in the therapy.

**Figure 3:**
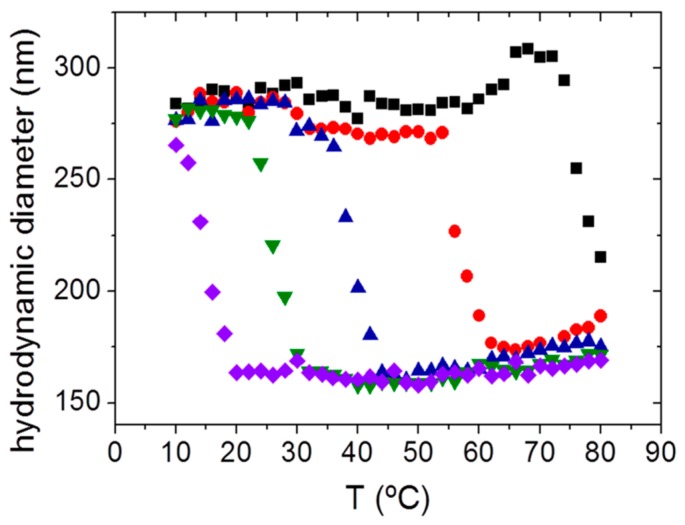
Average hydrodynamic particle size as a function of temperature at different pH and in a buffered solution (ionic strength of 150 mM). pH 6.0 (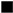); pH 6.5 (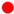), pH 6.9 (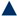), pH 7.5 (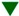) and pH 7.9 (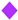). (Reprinted with permission from reference [25]. Copyright 2014 Wiley).

Dual stimuli-sensitive NGs were synthesized by emulsion polymerization employing PDEAEMA as main thermo- and pH-responsive polymer and ethylene glycol dimethacrylate (EGDMA) as cross-linker. Besides pH of the solution, ionic strength of the medium determines the swelling behavior of these PDEAEMA-based NGs. According to PDEAEMA properties, an increase in pH of the solution led to a reduction of the VPTT showing values physiologically relevant (38 °C and pH 7.1) (Figure 3) [25]. On the other hand, pH- and thermo-sensitive NGs based on PNIPAM and hydroxyethyl acrylamide (HEAA) were loaded with the antitumoral drug Paclitaxel (EE close to 72%). Regarding HEAA monomer, at acidic pHs amine groups are protonated leading to an increase in size of the NGs and subsequently, swelling and drug release are favored [71].

The introduction of disulfide-functionalized linkages to use redox potential for releasing the drug and controlling the biodegradability of the NGs can be combined with pH-responsive polymers to enhance drug targeting. Biocompatible polymers based on alginate were functionalized with MAA and a cystamine derivative as cross-linker to provide a pH/redox dual responsiveness. Dox was efficiently loaded and the release was dependent on both pH and presence of a reducing agent [72]. On the other hand, NGs with a dual pH/redox sensitivity based on the pH-responsive pentamethyldiethylenetriamine, cross-linked with a cystamine derivative and Dox-loaded, enhanced cell inhibition in human colon carcinoma cells HT-29 [73].

As discussed, Dox has been successfully included in several kinds of multi-stimuli NGs and one polymer used frequently is PVCL. PVCL functionalized-microgels with undecenoic acid (diameter size close to 250 nm and Dox EE about 40%) were synthesized by precipitation polymerization. Authors tuned hydrodynamic size, VPTT, and VPTpH by varying undecenoic acid concentration. At basic pH, carboxyl groups from undecenoic groups are negatively charged increasing the hydrophilicity and stability of microgel particles. As undecenoic concentration increases, the higher is the number of carboxyl groups and subsequently, hydrogen bonds between polymer chains and water augments resulting in an increase in VPTT. Accordingly, Dox-loaded microgels showed a slow release at physiological pH while the release was faster at a more acidic pH due to protonation-deprotonation behavior of undecenoic acid [74].

**Figure 4:**
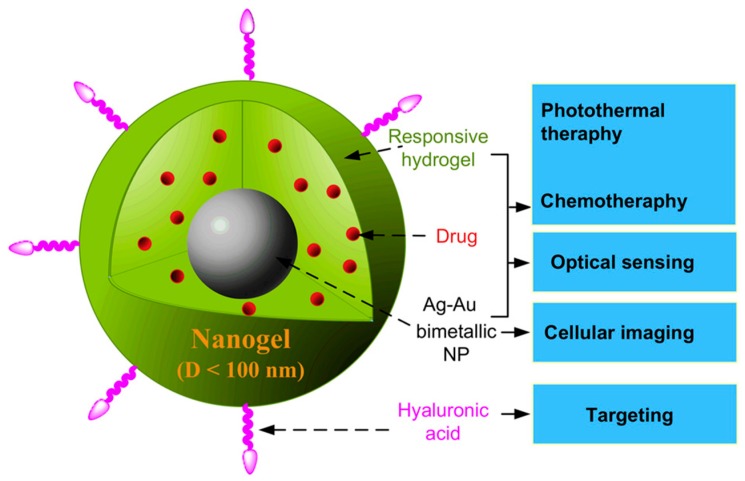
Schematic picture of multi-functional hybrid nanogel (NG) containing a bimetallic Ag-Au core coated with poly(ethylene glycol) (PEG) network and surface-decorated with hyaluronic acid (HA) molecules as targeting ligands. (Reprinted with permission from reference [76]. Copyright 2010 ACS).

The presence of magnetic NPs in pH- and thermo-responsive NGs has also been analyzed with promising therapeutic outcomes, specifically when these systems act as non-viral vectors [75]. Even, as previously discussed, a single nanocarrier can include magnetic NPs, stimuli-responsive polymers, and targeting ligands able to respond to various stimuli at the same time (Figure 4).

Aguirre et al. synthesized cationic and biodegradable PVCL&PDEAEMA-based core-shell nanogels using dextran-based macro-cross-linkers by means of a batch seeded emulsion polymerization process. Polyplexes were formed with these NGs and siRNA, taking advantage of the electrostatic attractions between the positively charged core-shell NGs and the negatively charged siRNA in simulated physiological conditions. siRNA release from these NGs was dependent on the pH of the release medium and the cross-linking density of the PVCL shell. These core-shell biodegradable NGs, responsive to pH and temperature changes, provide novel possibilities as gene delivery carriers [77].

## 3. Technological Aspects of Nanogels

To be considered as a real possibility for biomedical applications, NGs must maintain their properties and the payload both in aqueous media and after applying a technological process such as lyophilization and sterilization.

### 3.1. Encapsulation Efficiency and Drug Loading

Nanogels show a high versatility regarding encapsulation of bioactive substances since one system can include molecules with a different nature. Bioactive molecules can be incorporated in NGs by three main mechanisms: (i) physical entrapment; (ii) covalent interactions, and (iii) controlled self-assembly. Drug loading (DL) is often reported in terms of weight percent of the drug loaded per unit of nanogel while encapsulation efficiency (EE) is reported as weight percent of the drug encapsulated in the nanogel particles. An EE value between 5% and 25% is considered reasonable for nanosized drug delivery carriers [78] although NGs allow much higher drug loading (up to 50% of weight) [79].

Physical entrapment is one of the most employed procedures to encapsulate active substances into polymer network structures. Generally, drug loading occurs spontaneously in NGs avoiding drug exposure to any adverse conditions. NGs can be synthesized in the absence of the active compound and the drug loading process can be done efficiently later or even when the NGs are swollen and equilibrated in water (equilibrium swelling method), preventing the active agent losing its pharmacological activity. After reaching equilibrium, the bioactive is absorbed into the polymer network and DL is determined by the driving forces involving non-covalent interactions such as hydrophobic interactions and H-bonding between the active agent and the polymeric network. Moreover, cross-linking density in the network determines the EE (encapsulation efficiency) and DL [80].

Nanogels can encapsulate both lipophilic and hydrophilic compounds since their core-shell structure can include guest hydrophobic molecules in the hydrophobic core while aqueous agents can be located in the surface of the polymeric network. In the case of hydrophobic molecules, especially poorly soluble anticancer drugs, NGs increase the drugs’ solubility and stability, thus improving their efficacy. For example, monomethyl oligo(ethylene glycol) acrylate (OEGA) an ortho ester-containing acrylic monomer, and 2-(5,5-dimethyl-1,3-dioxan-2-yloxy) ethyl acrylate (DMDEA) using bis(2-acryloyloxyethyl) disulfide (BADS) as a crosslinker were used to synthesize temperature- and pH-sensitive NGs. Hydrophobic drugs such as Placitaxel and Doxorubicin (Dox) were loaded in this polymeric network by interactions with the hydrophobic ortho ester units from the copolymers. In addition, heating above VPTT promoted dehydration of the OEG chains resulting in a more hydrophobic nature and thus, improving encapsulation efficiency of both drugs, but especially Placitaxel which has a more hydrophobic nature [81].

Nevertheless, active substances can also be loaded in NGs during synthesis taking advantage of the easy, simple, and organic solvent-free process that helps maintain the pharmacological activity of the active compounds resulting in covalently bonded systems [82].

On the other hand, charged molecules such as siRNA or DNA, can establish electrostatic interactions with positively charged functional groups from NGs resulting in the formation of polyplexes and improving bioactive stability after encapsulation [68]. For example, after loading siRNA, dextran-based NGs showed a charge reversal from positive zeta potential values (+21.1 mV) to negatively charged polyplexes (−20.1 mV) [83]. Even, hemocompatibility studies demonstrated that negatively charged dextran-based polyplexes modified with poly(ethylene glycol) (PEG) showed minimal interactions with human blood cells “in vivo” [84].

After administration, NGs can safely carry the payload “in vivo”, move within the cells and release the contents in the desired place.

### 3.2.Sterilization of Nanogels

While developing new controlled drug delivery systems, sterilization of these vehicles is becoming especially important. Currently, several researches are focused on the effect of sterilization on both drug and carrier [85,86,87,88]. According to Pharmacopeias, the objective of sterilization is to destroy or eliminate unwanted living micro-organism contamination providing a Sterility Assurance Level (SAL) equal or better than 10^−6^ (probability of finding a non-sterility unit of less than 1 in 1 million). Sterilization of NGs designed for biomedical purposes must also be considered, since parenteral administration is one of the most common dosage routes for anticancer therapy.

The sterilization methods currently used for pharmaceutical products are dry and moist heat, chemical cold sterilization that employs ethylene oxide and other gases, filtration, and radiation. Sterilization of the final formulation is the most employed method. Nevertheless, if the pharmaceutical product cannot be sterilized during fabrication, aseptic processing will be required with the special complications derived from handling and transfer of materials in a sterile environment [89].

Among the variety of sterilization methods, membrane filtration is a useful procedure for sterilization of light or heat sensible nanosystems without adverse effects for the nanosystem properties [90]. However, in the case of particles with a broad size distribution, sterilization by filtration might not be the most adequate method because membrane pore size cannot be tailored for filtration particles of different sizes [91]. In addition, rigid or inflexible particles can lead to clogging of the sterilization membranes [92]. Despite the flexibility of nanogel particles, solid content loss can occur when passing through the standard filtration sterilization membranes (0.22 µm pore size).

When the nature of the active ingredient and its delivery system allows it, autoclaving is a non-toxic and safe for the environment method to sterilize. As previously described, autoclaving is a technique also used for NGs formation. The effect of high-temperature sterilization on a covalently linked system based on a protein, methemoglobin (Hb), and poly(acrylic) acid (PAA) was analyzed by activity and circular dichroism studies. The optimal PAA (450,000 MW) concentration to form nanogel networks was observed to be 0.2%. After sterilization in an autoclave (120 °C, 1.03 bar, 45 min), the peroxidase-like activity was evaluated in (i) free Hb; (ii) a physical mixture of Hb and PAA and (iii) the Hb-PAA network. Results showed a decreased peroxidase-like activity after sterilization with no significant differences among the three samples. In contrast to thermal denaturation, the pH-responsiveness was improved in the Hb-PAA nanogels with a narrower pH range than in the case of free Hb and the mixture Hb/PAA [93]. Although there are other studies in which NGs are sterilized by autoclaving [94,95], more data related to their stability before and after sterilization are necessary to determine the effect of heat in these nanosystems.

Radiation technique has become a useful method for “in situ” preparation and final sterilization of pharmaceutical products. The irradiation doses for biomedical applications are between 10–30 kGy depending on the size of the product. Irradiation doses are chosen according to the pharmaceutical product, the initial bioburden, SAL and radiosensitivity of the microorganisms [96,97]. Sterile pH-responsive and reproducible NGs composed of poly(vinylpyirrolidone) (PVP), PAA and oligonucleotides have been obtained by beam irradiation (40 kGy) by several research groups [98,99,100]. These NGs have shown absence of toxicity “in vitro”, and the ability to cross cellular membranes and reach the perinuclear area of the cytoplasm is potentially useful for biomedical applications [101]. pH-responsive NGs were prepared by gamma radiation-induced polymerization of acrylic acid (AA) in an aqueous solution of PVP. Gamma radiation (20 kGy) promoted hydrogen-bonding interactions between the AA molecules (proton donating carboxylic groups) and PVP (proton-accepting non-ionic polymer). Particle size, swelling behavior and physic-chemical properties, such as viscosity, depend on dose and exposure to irradiation temperature as well as on atmospheric composition during the process. Furthermore, PVP molecular weight and total concentration of the sample are directly related to the formation and mean size of polymeric nanoparticles. For example, nanogel particles were able to swell from 83 nm at pH = 4 to 446 nm at pH = 7 when a 1.5% mixture of PVP (1,300,000 Da)/AA in a molar ratio 35/65, at 35 °C and 20 kGy was used during the procedure. Regarding gamma radiation, it was observed that the higher the irradiation dose, the higher the cross-linking degree and the lower the swelling behavior of the nanogel particles [102].

Taking into account the key role of sterilization in intravenously administered NGs, further studies to determine the unwanted effects of the sterilization methods on the structural changes of polymeric NPs and the active ingredient included are required as well as the development of strategies to overcome these drawbacks [103].

## 4. Biocompatibility and Biodegradability in Nanogels

As model nanocarriers, NGs must remain chemically unaltered, protecting and releasing the active agents after reaching the target site. Likewise, essential properties of NGs are biocompatibility, not causing harmful side effects derived from their use, and biodegradability—the property of disappearing from the body once the active ingredient is released and its action is fulfilled.

### 4.1. Cytocompatibility Assays in Nanogels

Polymeric NGs synthesis implies using monomers, initiators, and surfactants that can have toxic characteristics. To provide information about their biocompatibility and the most safe and effective concentrations, “in vitro” studies are necessary [104,105,106,107].

Cell viability is analyzed using tests such as 3-(4,5-dimethylthiazol-2-yl)-2,5-diphenyltetrazolium bromide (MTT) assay, AlamarBlue, and trypan blue analysis. The MTT test, one of the most employed techniques, is a colorimetric test based on the selective ability of viable cells to reduce MTT into an insoluble compound, formazan.

As nanogels are being developed mainly as nanocarriers for cancer therapy, carcinoma cell lines are commonly employed in the cytocompatibility and cytotoxicity studies. For instance, lung cancer cells A549 were employed to test the toxicity of core-shell NGs based on linear PEG and/or non-linear polymers with oligo(ethylene glycol) (OEG) side chains by the MTT technique. Besides the good stability in physiological media and the ability to overcome freeze-thawing processes due to the presence of the side chains of OEG, these nanogel particles showed good “in vitro” tolerance in the range of concentrations from 0.5 mg/mL to 2 mg/mL. The lowest cell viability, close to 60%, was observed with the linear PEG-based NGs, without OEG shell at 2 mg/mL, probably due to their different morphology, less prone to hydrolysis [108].

Multifunctional biodegradable chitin-based NG were developed by controlled regeneration chemistry followed by sonication with an average diameter size <100 nm. These pH-responsive NGs were conjugated with QDs for drug delivery with simultaneous imaging and biosensing. MTT studies showed that these chitin-based NGs had no toxicity in six different cell lines: L929 (mouse fibroblast cell line); NIH-3T3 (mouse embryonic fibroblast cells); KB (oral cancer cell line); VERO cells (kidney epithelial cell line of the African Green Monkey); MCF-7 (human breast cancer cells), and PC3 (prostate cancer cell line). Furthermore, chitin-based NGs were distributed into cytoplasm and perinuclear region of cells and showed good hemocompatibility [109].

Cytotoxicity studies in NGs composed by OEG and pyridylsulfide encapsulating Dox, with different cross-linking densities, were performed by Thayumanavan and co-workers [97]. Dox-loaded NGs showed cytotoxicity in MCF-7 cells after 72 h, although these cytotoxicity values were lower compared to free Dox. Furthermore, cell uptake showed a significant accumulation in the cytoplasm and was significantly improved when Dox was loaded in NGs.

**Figure 5:**
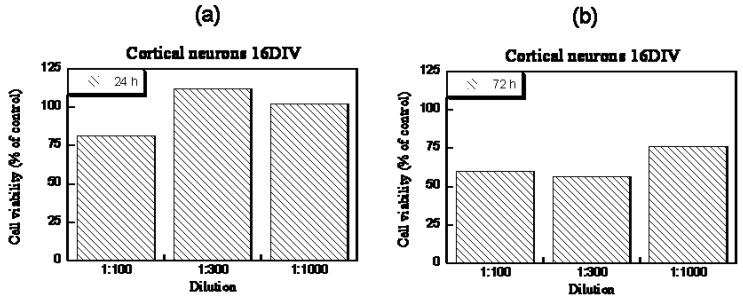
Cell viabilities of 16-day “in vitro” cultured rat primary neuronal cells after incubating for (**a**) 24 and (**b**) 72 h at three concentrations of nanoparticles (1%, 0.3%, and 0.1%). (Reprinted with permission from reference [110]. Copyright 2010 Wiley).

Imaz and Forcada [110] synthesized NGs based on the biocompatible polymer PVCL. The main monomer, VLC (*N*-vinylcaprolactam), was copolymerized with a sugar-based comonomer, 3-*O*-methacryloyl-1,2:5,6-di-*O*-isopropylidene-a-d-glucofuranose (3-MDG). These NGs had VPTTs that ranged from 31 °C to 36 °C. The biocompatibility of the synthesized NGs was analyzed in embrionary rat neuronal cells by fluorescence measurements at different particle concentrations (1%, 0.3%, and 0.1%) for 24 and 72 h. NGs had good “in vitro” tolerance with cell viabilities higher than 80% when cells were exposed for 24 h to 0.3% and 0.1% particle concentration. As expected, toxicity increased as contact time increased (Figure 5).

Core-shell NGs based on PNIPAM were conjugated with a surface peptide, erythropoietin, producing hepatocellular A2 receptor (EphA2) to target ovarian cancer. NG (about 54 nm mean diameter size) efficiently encapsulated siRNA (EE of 93% ± 1%). Targeted-nanogels showed good cell viability in two ovarian cancer cell lines, Hey and BG-1 (analysis performed by trypan blue and Tox8 assays). Cell uptake was also determined in (a) Hey cells that had a high expression of EphA2 receptor and (b) BG-1 cells which had a low one. Uptake of NGs was EphA2 receptor expression-dependent with a higher fluorescence levels in Hey cells compared to BG-1 cells. Additionally, authors observed that while naked siRNA was unable to penetrate cell membranes, gene silencing was efficiently achieved after adding peptide-functionalized NGs, suggesting that NGs play a protective effect and avoid siRNA degradation/denaturalization during cellular processes thus leading to an enhanced therapeutic effect [48].

More recently, thermo-responsive PVCL-based, pH-responsive PDEAEMA-based, and dually thermo- and pH-responsive PDEAEMA/PVCL-based core-shell NGs were synthesized using different dextran-methacrylates as macro-cross-linkers that provided biocompatibility and biodegradability properties. Dox was encapsulated in NGs by electrostatic interactions with high efficiency. “In vitro” studies in two cell lines; cervical cancer cell (HeLa) and breast cancer MDA-MB-231, showed that bare NGs were non-toxic and the toxicity of Dox loaded-NGs was lower than that of free Dox. To evaluate the responsiveness to environment changes, Dox release from NGs was analyzed at an acidic pH (5.2) and at neutral pH (7.4) with promising results. On the other hand, cellular uptake studies exhibited that Dox-loaded NGs were located at endosomes or lysosomes and Dox molecules were gradually released to the cytoplasm. These NGs were proved to meet the requirements for an ideal nanocarrier in HeLa and MDA-MB-231 cell lines [26].

### 4.2. Biocompatibility Studies in Animals

Taking into account the “in vitro” biocompatibility and cytotoxicity results obtained, the next step for nanogels to be considered as suitable nanocarriers is to display good “in vivo” tolerance [111,112,113,114].

Tamura et al. [18] synthesized by emulsion polymerization NGs based on PEG and EGDMA and determined their feasibility as nanocarriers for systemic administration. The cross-linking degree in PEGylated NGs varied depending on the EGDMA feed concentration (1, 2, and 5 mol %, in terms of cross-linking density. Size, zeta potential, and swelling ratio were dependent on pH and cross-linking density, as expected. The higher the cross-linking density the lower the swelling ratio due to the highly cross-linked structure of the polyamine cores that limit the water uptake. By cytotoxicity studies, PEGylated NGs with the highest cross-linking degree showed the lowest cytotoxicity in colon cells. Then, biocompatibility was evaluated in healthy mice after intravenous injection. The acute toxic effect derived from the polyamine cores of the PEGylated NG decreased from a median lethal dose (LD_50_) of 20 mg/kg with a cross-linking density of 1 mol % to >200 mg/kg when the cross-linking density increased to 5 mol %.

From a technological point of view, dextran is a saccharide widely employed in drug development processes, as it is a molecule approved for medical use by the FDA. Dextran based-nanogels have been evaluated in terms of biosafety with good results after IV administration [83,115]. The acute and long-term toxicity of fluorescent dextran-PAA based-NGs (of about 180 nm) were assayed after intradermal injection in mice. Biomarkers related to liver and kidney functions were analyzed from blood mice 1 and 30 days after NGs injection. Blood biochemistry did not show any difference in the indices studied (total protein, albumin, globulin, alanin transaminase, urea, etc.) and NGs were eliminated by urine in a few days. Nevertheless, there was a passing increase of the proteins alanin transaminase and aspartame transaminase, involving transient and slight liver damage 24 h after injection. Animal body weight, behavior, and clinical signs did not change significantly in the treated group compared with the untreated [116].

An “in vivo” tolerance study was performed in healthy volunteers that applied themselves by a cutaneous route, a Poloxamer 407 (PEG-PPO-PEG) thermo-responsive nanogel loaded with paromomycin. Previous to biosafety studies, paromomycin-loaded NGs showed suitable properties to be administered on the skin “in vitro”. Paromomycin-loaded NGs had a size close to 30 nm, an acidic pH similar to the skin (pH 5.8–6.3), a thermo-responsive pseudoplastic behavior (viscosity decreases when temperature increases) and a proper spreadability. Cytotoxicity assays in RAW and VERO cells demonstrated a good cytocompatibility (cell viability ≥ 80%). “Ex vivo” measurements in mice and pig ear skin confirmed two-times more antileishmanial effects in the paromomycin-loaded NG than the paromomycin solution. After that, volunteers with healthy skin applied paromomycin-loaded NGs once a day for 3 days in a controlled ambient chamber (25 °C and 45% of relative humidity). Then, abnormalities in the epidermal permeability barrier structure were assayed by transepidermal water loss measurements with no significant differences between the paromomycin-loaded NGs and the untreated control sites, demonstrating that this poloxamer-based nanogel had an appropriate “in vivo” tolerance. Furthermore, this formulation may be consider as a potentially useful system for leishmaniosis therapy requiring a dose almost 20 times lower than in the currently topical treatments [117].

Biosafety and biocompatibility analysis in animals are of great importance and although they are becoming more present, further “in vivo” tolerance studies are necessary, particularly in humans, to allow verification of the effectiveness of these systems “in vivo”, thus promoting clinical studies.

### 4.3. Biodegradation Mechanisms in Nanogels

Degradation of polymeric networks depends on the polymers as well as the cross-linking agents employed during the synthesis of NGs. Network structure can be disintegrated by incorporating labile cross-linking points into the NGs, yielding freely soluble polymer chains. In this sense, biodegradation mechanisms can be governed by enzymatic activity and physiological/external changes.

**Figure 6:**
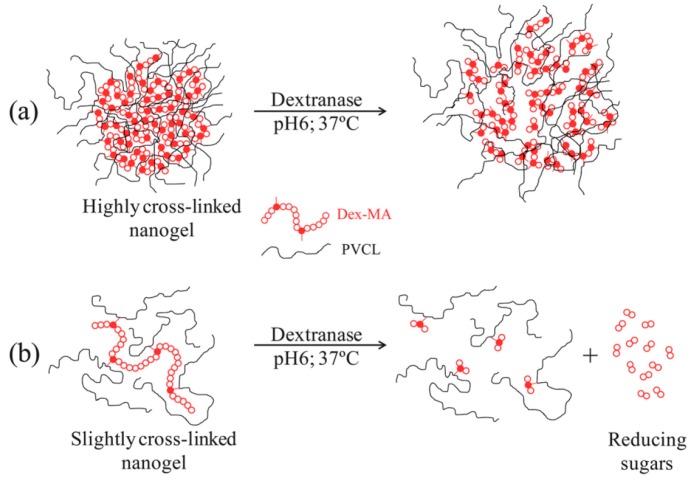
Degradation mechanisms for (**a**) highly cross-liked nanogel synthesized with a high degree of substitution and high molecular weight dextran-methacrylate macro-cross-linker and (**b**) slightly cross-linked nanogel synthesized with a low degree of substitution and low molecular weight dextran-methacrylate macro-cross-linker. (Reprinted with permission from reference [120]. Copyright 2013 RSC).

An interesting approach is the synthesis of enzymatically degraded NGs. The enzymes responsible for their degradation can be included in the polymeric network. After enzyme uptake in the inactive form by NGs, the enzymes are activated under physiological conditions leading to degradation of the network [118,119]. Aguirre et al. [120] synthesized enzymatically degradable PVCL-based NGs by batch emulsion polymerization using dextran-methacrylate derivatives as macro-cross-linkers. Dextranase, an enzyme that degrades dextran by cleaving linkages between the glucose monosaccharide’s that compose dextran polysaccharides, was absorbed into the NGs efficiently (95% of enzyme uptake using 100 U dextranase/g NG) in the inactive form (pH = 8 and 20 °C). Then, the enzyme was activated by increasing temperature (37 °C) and acidifying the media (pH = 6). Enzymatic degradation was governed by the degree of substitution (number of methacrylate groups per dextran chain) and dextran molecular weight of the macro-cross-linker used during NGs synthesis. In the case of slightly cross-linked nanogel particles (low degree of substitution and low molecular weight macro-cross-linker) dextranase released reducing sugars from dextran chains causing de-swelling of nanogel particles. Otherwise, highly cross-linked particles (high degree of substitution and high molecular weight macro-cross-linker) were swollen. In this case, dextranase was not able to release reducing sugars due to the high cross-linking density of the polymeric network, but cleaved some glucopyranosyl bonds modifying the network structure (Figure 6).

On the other hand, network disintegration can appear when external stimuli such as a significant difference in the pH or in the redox potential, enzymatic activity and even light irradiation are applied [121,122,123,124]. In many cases, NGs degradation triggers the release of the active ingredient incorporated in the polymeric network [125,126,127]. As previously discussed, pH in healthy cells is higher than in tumor cells and, this difference is also evident between endosomal and extracellular pH environments. As occurs with the pH, GSH concentration in cytoplasm is higher than in extracellular environment and similarly, the redox potential is also increased in some cancer types [32]. To take advantage of these tumor tissues properties, several works have been aimed at the development of stimuli-responsive NGs that can release the active ingredient by degradation, mimicking the intracellular/tumor cells environment [128,129]. Redox/pH dual stimuli NGs, using MAA as main monomer and *N*,*N*-bis(acryloyl)cystamine as disulfide-functionalized cross-linker, were prepared by distillation-precipitation polymerization. NGs had a unimodal size distribution (240 nm in the dried state) and entrapped Dox by electrostatic interactions with a high loading efficiency (95.7%). Degradation behavior of these NGs was monitored by turbidity and GPC measurements. After the addition of a reductive agent, dithiothreitol (DTT), the turbidity of the NGs dispersion lead to a clear solution within 30 min and the degraded polymers had a low molecular weight. Under acidic pH (pH = 5) and at different GSH concentrations, 2 mM, 5 mM, and 10 mM, Dox release increased from 57%, 78%, and 95% respectively in 24 h. Otherwise, at pH = 7.4 and without reducing agents, mimicking blood circulation, Dox release from NGs was about 15% in 24 h indicating a low drug leakage in physiological pH conditions and decreased systemic toxicity. In addition, Dox-loaded NGs efficiently killed glioma tumor cells [72]. Regarding the enzymatic activity, Dox was also loaded into pH-responsive NGs based on chitin and poly(caprolactone). Degradation was studied in the presence or absence of lysozyme, an enzyme that cleaves the glycosidic linkages of chitin. After 30 days, around 80% and 70% of Dox-loaded chitin-poly(caprolactone) NGs were degraded with and without lysozyme, respectively. The pH-responsive behavior was supported by “in vitro” studies in which Dox release was significantly faster at acidic pH compared to physiological conditions. Glioma cancer cells uptake Dox-loaded NGs, and Dox release took place in the cytoplasm according to the acidic pH and the reduced intracellular microenvironment of glioma cancer cells [130].

Nanogels are also being developed as non-viral gene vectors due to their capacity to protect the active agent from degradation and release it in a controlled fashion. Following the approach of releasing the cargo as a consequence of NGs degradation, the vesicular stomatitis virus matrix protein gene was loaded into PEI nanogels. To reduce the PEI high toxic effect, short PEI chains were coupled into a longer one using heparin as biodegradable cross-linker, thus developing biodegradable NGs by amide bond formation between the amine groups of branched PEI and the carboxyl groups of heparin. The biodegradable cationic NGs showed a mean particle size and zeta potential of 75 nm and 27 mV, respectively. Biodegradation was studied “in vivo” after IV injection of heparin-PEI NGs in rats. Twenty-four hours after administration, NGs were excreted through urine in the form of low molecular weight PEI chains due to the quick degradation of heparin by physiological enzymes. Indeed, these nanosystems efficiently inhibited colon cancer cells and the growth of colon carcinoma “in vivo” [131].

However, biodegradation can be hampered in some types of NGs due to the covalent bonds established between polymer segments according to the synthesis process employed [132].

## 5. Biotechnological Uses of Nanogels

Design and production of new nanogels based on more complex nanostructures is not only dependent on a deeper knowledge of how these nanocarriers interact with biological systems, but also on the requirements established by the specific biotechnological applications. Rapid developments occur almost on a daily basis and they have been directed to achieve new nanogels for controlled and targeted drug delivery for the treatment of several diseases. Main research works involving NGs as therapeutic carriers for active substances are collected in Table 1.

### 5.1. Nanogels for Cancer Therapy

The high incidence, rapid evolution and the high mortality derived from cancers are the main causes of developing novel therapies to improve traditional tumor treatments. The evolution in the knowledge about proteomic [133], therapeutic targets [134], improved diagnostic strategies, and emerged therapies [135] have led to a hopeful reduction in the cancer rate incidence [136].

**Figure 7:**
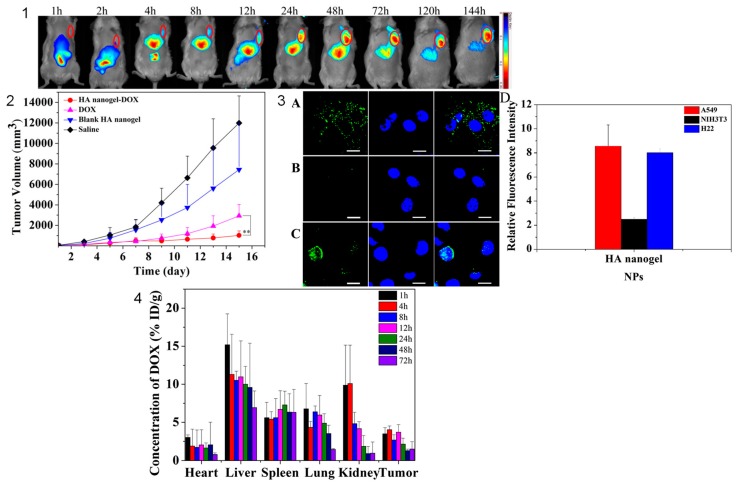
(**1**) In vivo near-infrared (NIR) fluorescence imaging of fluorescent Dox-loaded HA-based NG IV administered in induced tumor mice by injection of H22 tumor cells. The fluorescence intensities are represented by different colors according to color histogram. Red circles on the images display the localization of the tumor; (**2**) Tumor growth inhibition in H22 tumor-bearing mice that received as treatment Dox-loaded HA-NGs, free Dox, unloaded HA-NGs and saline solution; (**3**) Uptake of Dox-loaded HA NGs by cells with high CD44 and CD168 receptor expression (**A**) A549 and (**C**) H22 and low receptor expression cells (**B**) NIH3T3. Fluorescent HA NGs are green-colored in the confocal laser scanning microscope images indicating uptake by HA-receptor cells. The scale bar = 10 μm, (**D**) Flow cytometry analysis of HA NGs incubated with A549, NIH3T3 and H22 cells for 4 h; (**4**) Biodistribution of Dox for Dox-loaded HA NGs in H22 tumor-bearing mice at various time points after intravenous injection. (Reprinted with permission from reference [147]. Copyright 2015 Elsevier).

Among these approaches, nanosized carriers are considered to be one of the most promising strategies. Nanogels are becoming popular among other DDS to overcome the drawbacks in tumor therapies due to the ability of stimuli-responsive polymer-based NGs to respond to external changes. In addition, as previously discussed, surface-decorated NGs enhance drug therapeutic effects [137,138]. So, NGs have been prepared to passively (EPR effect) and actively target tumor cells/tissues/organs showing promising results “in vitro” and “in vivo” [63,81,139]. Dox is one of the most employed drugs to study the suitability of the carrier. In fact, several formulations based on Dox-loaded LPs are currently on the market [140]. It is worth noting that Thermo-Dox, an intravenously injected formulation based on temperature-sensitive LPs loading Dox, is in clinical trials [141]. According to Thermo-Dox specifications, an external heat source must be applied to produce changes in the LPs structure and create openings that release Dox directly in the target tumor. Phase III OPTIMA trial, approved by the FDA, will evaluate the efficacy of Thermo-Dox LPs combined with hyperthermia therapy in primary liver cancer [142]. Following this research line, Dox has been also loaded in NGs to achieve a controlled release in the target site according to triggered stimuli from tumor cells/tissues [143,144,145,146]. For example, Yang et al. [147] developed HA-based NGs to target cancer cells since CD-44 and CD168 receptors (natural receptors for HA) are overexpressed in many tumors. By methacrylation strategy, HA was functionalized and linked to di(ethylene glycol) diacrylate (DEGDA) by radical copolymerization to obtain polymeric NGs with a stable mean diameter size (78 ± 2 nm). This nanosystem showed a pH-dependent zeta potential value, being strongly negative at neutral pH (−45.0 mV) and increasing on acidifying the medium (−12 mV at pH 4.2). HA-based NGs were enzymatically degraded by physiological enzymes, hyaluronidases and lipases. In combination with enzymatic activity and acidic pH, Dox loaded with high efficiency in the NGs network (close to 62%), is released in a faster fashion than in neutral pH and in the absence of enzymes. Cells with high CD44 and CD-168 receptor expression (A549 and H22 cells) showed a higher Dox uptake than NIHT3T3 cells (low CD44 and CD168 expression) suggesting a receptor-dependent cellular uptake. After IV injection in H22 tumor-bearing mice, “in vivo” near-infrared (NIR) fluorescence imaging technique pointed out that Dox-loaded NGs are accumulated at the tumor site. This accumulation effect was visible for at least 144 h post-injection. Finally, Dox-loaded HA-based NGs efficiently inhibited tumor growth in mice (91% of tumor growth inhibition in animals treated with Dox-loaded NGs versus 75% in animals treated with free Dox) due to (i) the higher uptake by tumor cells and (ii) the superior Dox accumulation in the tumor site (Figure 7).

**Figure 8:**
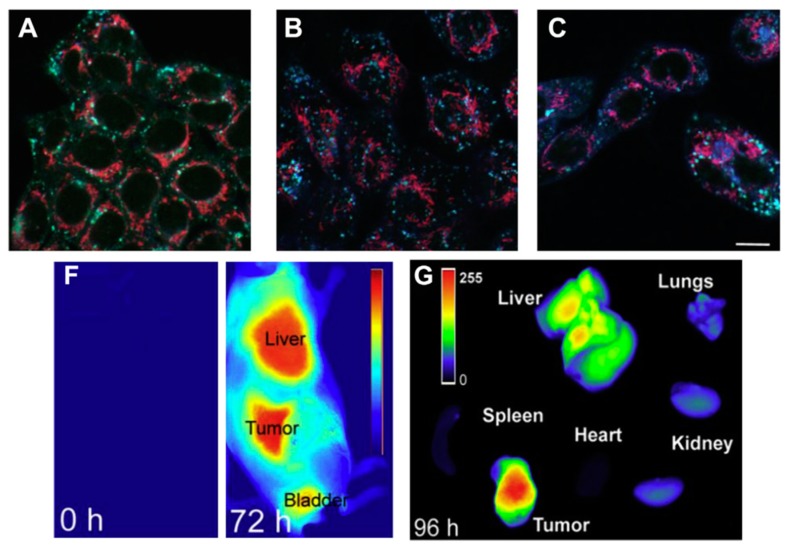
Co-localization of Pc4 loaded MBA-functionalized triple responsive expansile NGs (TRN) (red) in lysosome and mithocondria at 2, 3 and 21 h respectively for (**A**); (**B**) and (**C**). Scale bar in (**C**) is 10 µm. (**F**) Biodistribution of MBA-Pc4-TRN before and 72 h after injection in induced tumor mice; (**G**) “Ex vivo” images 96 h after MBA-Pc4-TRN according to color scale. (Reprinted with permission from reference [148]. Copyright 2014 Elsevier).

Taking advantage of the coexistence of an increased temperature and the differences in pH and redox gradients that appear in cancer cells or tissues, multi-functional NGs (responsive to temperature, pH, and reducing ambient) have been designed to enhance therapy in tumor diseases. In combination with this approach, designing targeted NGs led to a programmable drug release at the specific target site. In that sense, He et al. [148] synthesized triple responsive expansile NGs (TRN) sensitive to temperature, pH, and reducing ambient. A copolymer of poly[(2-(pyridin-2-yldisulfanyl)-*co*-[poly(ethylene glycol)]] (PDA-PEG) reacted with PNIPAM by free radical polymerization and copolymers were modified by adding cysteamine (that provides responsiveness to redox potential). In addition, authors also targeted this TRN by adding 4-methoxybenzoic acid (MBA), a specific ligand for sigma-2 receptor that is present in head and neck tumor cells, among others. TRN behavior was unexpected because instead of shrinking, particle size remarkably expanded when temperature increased from 30.5 to 47 °C and MBA cross-linking density increased from 20% to 40%, respectively. Likewise, particle size of TRN was pH-dependent showing an expanded size upon the decrease of the pH from neutral to lysosomal values. Significantly, after 2 h of exposure to cytosol reducing conditions (10 mM DTT) at body temperature, nanogel particles swelled from 108 to 1200 nm. To study the ability of this TRN as a novel approach for cancer therapy the silicon phthalocyanine photosensitizer, Pc4, was loaded into the TRN. Pc4 is a highly hydrophobic molecule that can kill cancer cells by dividing the mitochondria. However, to avoid toxicity and adverse effects and increase drug efficacy, releasing Pc4 specifically to the mitochondria of cancer cells is mandatory, including Pc4 into the network at 30% of cross-linking MBA density, the VPTT of the TRN dropped to 37 °C. “In vitro” Pc4 release rate from TRN was faster at acidic pH (30.7% Pc4 released in 24 h at pH 5.0 compared to 13.6% after 3 days in buffer solution at pH 7.4) suggesting that size enlargement correspond with a quick drug release. Confocal images assured that MBA-targeted TRN were taken by cells expressing sigma-2 receptor via endocytosis and TRN expanded inside cell lysosomes. In a mice model of head and neck tumor, MBA-Pc4-TRN was retained in tumor sites and liver, 72 h after vein tail injection. However, 96 h post-injection liver showed much less fluorescence while tumor sites still retained the luminescence (Figure 8).

In addition, NGs, as other systems, can include not only the active substance, but also an imaging agent combining dual effects, therapeutic and imaging, in one carrier [7,170,171]. This feature is essential in the treatment of cancer, especially to improve therapies avoiding harmful side effects. Noteworthy, the “in vivo” localization of nanocarriers allows determination of the efficacy of the therapy and modifies it according to the specific patient requirements. Taking advantage of the physico-chemical drug properties, the increased temperature of local media (cancer cells) and external heat and light sources, and specific receptor binding, a HA-based nanogel containing graphene (an efficient agent in killing tumor cells) and Dox conjugates was developed. Functionalized graphene linked Dox by ester linkages, and the conjugates were coated by disulfide-cross-linked HA molecules. Almost 100% of Dox was released in 48 h when NGs were exposed at pH 5.0, 10 mM GSH concentration, and laser irradiation, responsible for initiating a single oxygen generation from graphene that breaks disulfide bonds from HA network. “In vitro” and “in vivo” studies show a high efficacy of the HA-based NGs killing tumor cells and inhibiting tumor growth. HA-based NGs are uptaken via CD44-receptor by A549 cells and the “in vivo” assays display a combined therapeutic effect of graphene and Dox located specifically at tumor sites with no adverse effects on healthy organs due to the stimuli-responsive “multi-drug” release [149]. A similar approach in multi-modality was followed by Zhou’s group, developing several types of thermo-responsive NGs for drug release and imaging [172,173,174]. For example, a hybrid nanogel (<100 nm) based on PEG bonded to HA, as targeting molecule, and containing Ag-Au NPs (10 ± 3 nm) was synthesized by precipitation polymerization (Figure 4). The hybrid NGs efficiently encapsulated the antitumor drug temozolomide (from 35.2% to 46.5%) due to the formation of hydrogen bonds between the ether oxygens of PEG and the amide groups from the drug. An increased temperature produced a shrink of the network due to the transition from hydrophilic to hydrophobic PEG chains. As a consequence, temozolomide is expelled and diffused across the network in a temperature-dependent manner. In addition, these hybrid NGs emit strong fluorescence that allows a combined therapy based on temperature increase (i) of the local environment or (ii) caused by an exogenous stimulus (NIR irradiation) [76].

Cancer therapy can also be improved by reducing the frequency of administrations. For this purpose, the simultaneous delivery of Dox and methotrexate triggered by acidic pH and increased temperature was successfully achieved from superparamagnetic NGs based on copolymers of PNIPAM and MAA derivatives encapsulating iron oxide NPs (<30 nm) [150].

As result of several efforts and intensive research, the multi-stimuli responsive NGs have emerged with promising “in vitro” and “in vivo” results as novel platforms for antitumor agent delivery. In spite of the fact that the translational to clinical application is complicated, especially when synthesis and functionalization are complex and arduous, multi-responsive NGs enhance the efficacy of tumor therapy. Indeed, multi-functional stimuli-responsive NGs allow diagnostic and targeted drug release in a single carrier. Therefore, multi-stimuli NGs are remarkably attractive for developing innovative nano-medicines for cancer treatments.

### 5.2. Nanogels for Chronic Diseases

Diabetes is one of the most common chronic diseases and there is an increased demand for improving the therapeutic regimen, elaborating noninvasive glucose monitoring as well as finding new ways for insulin administration [175,176]. From a technological point of view, the development of glucose sensitive nanogels can overcome the challenges associated with diabetes treatment. Indeed, to achieve near normo-glycemia values, NGs can release insulin in a controlled fashion by glucose-responsive swelling/shrinking mechanisms [177,178].

Glucose-responsive systems can be divided into three classes: glucose oxidase (GOx), lectin, and phenylboronic acid (PBA)-modified systems. Briefly, GOx is an enzyme that consumes glucose to gluconic acid which causes a decrease in the pH of the microenvironment allowing the release of encapsulated agents [179]. Among lectins, Concavalin A (ConA) is the glucose-responsive carbohydrate more linked to polymer structures. In the presence of glucose, ConA is dissociated from the network to bind glucose and subsequently, active agents are released. Nevertheless, these systems have shown low stability during storage restricting the practical applications [180]. The last glucose-responsive system was based on polymers functionalized with PBA. PBA have two forms in equilibrium in water, one hydrophobic and a charged one. In the presence of glucose, this equilibrium is broken because the charge compound of PBA forms a reversible complex with glucose, and subsequently the release of the active agents takes place. NGs functionalized with modified-PBA have been widely studied for insulin release due to their good stability during storage and the high specificity of the PBA for glucose molecules [181]. Wu et al. [151] developed a nanosystem that integrates glucose detection and a self-regulated insulin delivery. These authors reported the synthesis of multifunctional NGs containing Ag NPs (10 ± 3 nm) in the core and a shell composed of poly(4-vinylphenylboronic acid-*co*-2-(dimethylamino)ethyl acrylate (p(VBPA-DMAEA)) as glucose-detection system. NGs (<200 nm in a swollen state) have a VPTpH close to physiological pH at 37.2 °C (size of about 120 nm, in the collapsed state). According to the authors, when NGs are in an enriched-glucose medium, PBA is dissociated to bind glucose allowing expansion of the polymer chains. As a consequence of the glucose-induced volume located at the shell of the nanogel, the optical properties in the Ag NPs core change, converting the glucose concentration modification in the local media into optical signals. This behavior is selective to glucose molecules and insulin release is determined by glucose concentration. “In vitro” studies have shown that the higher the glucose levels, the higher the insulin released from NGs. In addition, this mechanism is reversible and when glucose is removed from the media, NGs recover both their initial structure and fluorescence.

**Figure 9:**
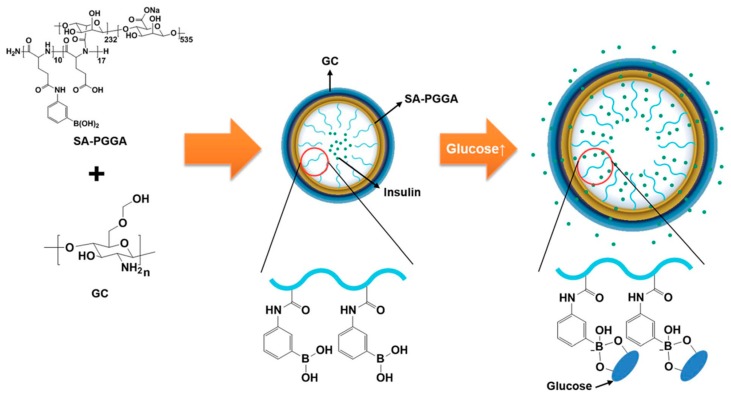
Schematic picture of glucose sensitivity of a double-layered nanogel composed by glycol chitosan/sodium alginate(SA)-poly(l-glutmate-*co*-*N*-3-l-glutamylphenylboronic acid) (PGGA) encapsulating insulin and releasing it in the presence of glucose by complexation between PBA derivative and glucose. (Reprinted with permission from reference [152]. Copyright 2015 RSC).

On the other hand, studies performed in diabetic animal models have demonstrated that NGs based on polymers functionalized with PBA derivatives can release insulin in a controlled fashion according to glucose blood levels. Actually, after administration, NGs can maintain low glucose levels for longer compared with free insulin [152,153] (Figure 9).

Additionally, pH-responsive NGs have been also proposed as an approach for insulin orally administered. At stomach pH (1.2–2) insulin release is significantly lower compared to the delivery at physiological pH due to the pH-dependent swelling/shrinking behavior of the polymeric nanosystems. These systems are composed of polymers such as hydroxypropylmethylcellulose/PAA (HPMC/PAA) and PEG-poly(aspartic acid), that exhibit a pH-responsive swelling behavior attributed to the ionization of the acid groups [154,155].

Recently, glucose-responsive NGs have proven to be suitable for tumor drug delivery. Liu et al. [156] synthesized superparamagnetic NGs sensitive to glucose, pH, and temperature. Stimuli-responsive hybrid NGs were based on poly(vinyl alcohol)-*b*-poly(*N*-vinylcaprolactam) (PVA-*b*-PVCL) copolymers and boronic acid functionalized γ-Fe_2_O_3_ NP via boronate/diol bonding. Tamoxifen, which has a hydrophobic nature, was loaded in the NGs and located in the hydrophobic domain of PVCL. Drug-loaded NGs (mean diameter size of 580 nm and drug loading efficiency close to 24%) controlled tamoxifen release at different pHs, glucose concentrations and after exposure to a magnetic field “in vitro”. Besides showing good cytocompatibility, tamoxifen-loaded NGs significantly enhanced inhibition on cell proliferation at acidic pH, close to tumor cells, and at increased glucose concentration in human melanoma cells. Taking into account the circadian rhythms, authors suggest that these nanosystems may be considered as an approach to improve therapeutic efficacy of orally administered drugs.

NGs have also shown a high potential for the treatment of pulmonary disorders. In a study performed by De Backer et al. [157], cationic NGs based on (2-[methacryloyloxy]-ethyl) trimethyalammonium chloride and dextran were allowed to complex with siRNA. After studying the interactions between the siRNA-loaded NGs and the negatively charged pulmonary surfactants, authors concluded that surfactants potentiate siRNA delivery from dextran NGs. These systems, administered with synthetic surfactants have been proposed as a novel option for the treatment of pulmonary diseases caused by surfactant dysfunctions. The effectiveness of these targeted NGs loaded with dexamethasone has been successfully evaluated for pulmonary inflammation in a mice model.

Biocompatible NGs composed of lysozyme and dextran were conjugated with Anti-ICAM-1, an antibody of the cell adhesion glycoprotein, ICAM-1, located in pulmonary endothelium. ICAM-1-NGs were found in a significantly higher concentration in lungs after IV injection compared to spleen and liver accumulation. In addition, pro-inflammatory cytokine levels in the lungs were reduced in the endotoxemic mice pre-treated with targeted NGs loaded with dexamethasone [158].

Huang et al. synthesized biocompatible NGs composed by PVA and F-127 (PEO-PPO-PEO triblock copolymer) by a self-assembling procedure. NGs encapsulated both magnetic NPs and ethosuximide, an anti-epilepsy drug. The thermo-responsive NGs had a 71 nm diameter size in the swollen state at 25 °C and 22 nm in the shrinkage state at 45 °C. By applying a magnetic field, NGs released ethosuximide instantaneously due not only to the shrinkage of the particles but also by the occurrence of disruptive changes in the structure as a consequence of the increased temperature after magnetic exposure. To test these nanosystems, rats suffering epilepsy were intravenously injected ethoxumide loaded-NGs and then received a high frequency magnetic field. Epileptic episodes were decreased by at least 60% after magnetic field exposure reporting promising results for epilepsy therapy [159].

These works open new opportunities in the field of the treatment of chronic diseases providing proof of concept that multi-stimuli responsive NGs lead to an improvement in therapeutic outcomes.

### 5.3. Nanogels for Neurodegenerative Diseases

Considering the progressive evolution in a continuously aging population, an effective therapy involving neurodegenerative pathologies, such as Alzheimer and Parkinson, remains a challenge. After systemic administration, the most important factor limiting the access of the drugs to reach the specific site of action is the blood brain barrier (BBB). Drug delivery in the brain is hampered by the structure of the BBB made up of highly differentiated cells with intercellular tight junctions that hinder the access of both small and high molecular weight molecules [182]. Although invasive methods effectively overcome the BBB, they carry a high risk for the patients and costs for hospital stays. In particular, invasive strategies include intracerebral injections, implants or drug delivery by disruption or alteration of the BBB causing neurotoxicity due to the entry of endogenous substances [183]. Non-invasive drug delivery strategies imply the nasal route, the hydrophobization of small molecules to passively cross through the BBB and the use of nanocarriers [184,185,186]. Progress in the development of novel particulate DDS resulted in enhanced nano-formulations that cross the BBB ensuring sufficient brain accumulation without administrating elevated drug doses [187,188]. In order to achieve a proper drug concentration in the brain, nano-formulations should be positively charged and decorated with brain-specific vectors to establish both electrostatic and specific interactions with brain endothelium cells [189]. In this sense, NGs have been successfully developed for the treatment of neurodegenerative diseases. For example, to enhance the permeability through the BBB, Soni et al. [160] hydrophobized 5-FU and, after that, loaded it in the hydrophobic core of nanogels based on PNIPAM and PVP and synthesized using *N*,*N*′-methylenebisacrylamide as cross-linker. NGs containing 5-FU had a diameter size of 50 nm and EE close to 80%. 5-FU-loaded NGs were coated with polysorbate 80 in order to lead the uptake by endothelial brain cells after interaction with the low-density lipoprotein receptor [190]. Mice received an injection of 5-FU-labeled NGs coated and uncoated with polysorbate 80. Radioactivity studies showed that at 1% of polysorbate 80, the maximum possible accumulation of NGs in the brain was observed. Nevertheless, the effect of polysorbate 80 is controversial. For example, methotrexate was loaded in NGs based on the natural polymer chitosan, and then, decorated with polysorbate 80. Pharmacokinetic studies in rats indicated an increased drug concentration in the brain when methotrexate-loaded NGs are injected compared with the free drug, although any additional effect, such as the amount of the drug delivered to the brain, comparing the surface-decorated or bared NGs was remarkable [161,162].

Sterile PVP-PAA NGs synthesized by gamma irradiation (diameter size 572 nm) were loaded with dopamine (EE, 72%). Dopamine-loaded NGs were administered by intraperitoneal injection in rats suffering reserpine-induced Parkinson disease. Dopamine was efficiently delivered from NGs across the BBB at an optimized dose (12 mg/kg) and consequently, the catalepsy state improved in the Parkinson induced experimental rats [163].

Vinogradov and co-workers [164] developed NGs of PEG and PEI and those that formed complexes with oligonucleotides (ODN) by electrostatic interactions (particle diameter size less than 100 nm). NGs were decorated with insulin and transferring molecules to provide brain-binding specificity. Besides being nontoxic, ODN were efficiently delivered from nanogels in an “in vitro” model of BBB. After intravenous administration in mice, ODN-loaded NGs were found to be accumulated in the brain by over 15-fold when compared with the free ODN, while free-ODN concentration was 2- and 3-fold higher in liver and spleen, respectively. Very recently, NGs decorated with a brain-specific peptide, BP2, were synthesized using biodegradable and biocompatible natural-based materials, polylysine and cholesterol for the treatment of Human Immunodeficiency Virus (HIV)-associated encephalitis and neurodegeneration. Cationic BP2-NGs (diameter size 32–35 nm) were intravenously injected in mice reaching a brain accumulation 2.5–3-fold higher than with non-vectorized NGs [165].

The development of these novel drug-loaded nano-formulations can be considered as the further step to adequate therapies for neurodegenerative diseases by decreasing the toxicity and improving efficacy by brain-specific accumulation.

### 5.4. Nanogels and Tissue Engineering

Pullulan is a polysaccharide industrially obtained from starch and is widely used in research due to properties such as film-forming and high compatibility among others. Pullulan can be modified by adding cholesterol moieties, and the functionalized molecules are able to form NGs (of about 30 nm diameter size) by self-assembly in water. The size and density of nanogel particles can be controlled by changing the degree of substitution of the cholesterol molecules in the NGs. Cholesterol bearing pullulan (CHP) NGs have a high biocompatibility and they can be used for bone regeneration purposes [191,192,193].

The potential of CHP NGs as guided bone regeneration was tested by Miyahara et al. [166]. Acryloyl-modified cholesterol-bearing pullulan NGs were mixed with thiol-modified PEG to form a membrane-shaped nanogel which was applied on the bone of rats that suffered from induced bone defects. The animal group treated with the CHP NGs-based membrane developed new bone instead of calcified areas and newly-formed bone appeared at an earlier time point compared to the collagen group (control group). According to the authors the particularity of these NGs is to store endogenous molecules, both hydrophilic and hydrophobic inside the network, and exchange them in a controlled fashion with the extracellular media. Furthermore, CHP NGs containing a lipophilic compound, prostaglandin E1, was tested in a rat model of skin defects with promising wound healing results [167].

Besides encapsulating hydrophobic molecules such as prostaglandins, CHP NGs can increase the stability of encapsulated peptides and protect them from aggregation “in vivo”. For example, W9-peptide accelerates bone formation “in vivo” [194]. W9-peptide is an antagonist of (i) the activator of nuclear factor-kB ligand (RANKL) and (ii) the inflammatory cytokine tumor necrosis factor (TNF)-a. These molecules are involved in inflammatory disorders and osteoclastogenesis. CHP NGs (1.4 cholesterol moieties per 100 anhydrous pullulan glucoside units) successfully complexed W9-peptide, leading to the formation of stable nanogel particles (of about 40 nm diameter size). In a mice low dietary Ca bone destruction model, subcutaneous injections of W9-peptide-CHP NGs inhibited bone loss suggesting the suitability of the CHP NGs as carriers for peptides [168]. Bone tissue engineering was also achieved by combining CHP NGs and two growth factors in a hybrid hydrogel network. The stability of the growth factors was improved inside the system and in addition, CHP NGs gradually released the bioactives, improving the healing and the osteo-inductive activity in the bone [169].

Previous studies have demonstrated that copolymerization of pullulan and responsive polymers lead to stimuli-responsive NGs with a high biocompatibility [126,129]. Nevertheless, more research is necessary to develop multi-responsive NGs as useful tools for innovative clinical applications in regenerative medicine.

## 6. Conclusions

The treatment for current diseases requires the development of systems designed to protect and deliver the active agent close to the target site in a controlled fashion. In this sense, NGs are emerging tools as suitable nanocarriers with a multifunctional and responsive nature for simultaneous imaging of tumors and drug delivery. NGs also show great use in gene therapy to protect the active substance improving the therapeutic effect. The design of NGs has been directed to obtain nanoparticles able to respond to environmental stimuli, such as a gradient in pH, temperature changes, ligand molecules, or even by applying an external factor as in the case of hybrid systems combining magnetic nanoparticles and stimuli-responsive NGs.

The tolerance of NGs intended for biomedical applications is a critical issue as NGs can travel throughout the body to target tissues. Polymers employed in the synthesis of NGs must have good tolerance, being biocompatible and biodegradable. Polymerization processes are daily evolving to obtain multifunctional and biocompatible NGs that can respond to environmental changes. The development of biocompatible NGs by using biocompatible polymers is also a critical point in reaching the clinic. In fact, there are a number of interesting nanocarriers under investigation with associated toxicity that prevents their translation to biomedical practice. To assure elimination as well as a high tolerance, several NGs have been synthesized with biocompatible and biodegradable materials. Furthermore, according to the great number of works regarding multi-stimuli NGs in recent years reported in the literature, it is encouraging to expect important progress in translational research focused on using NGs in biomedical applications. Following this line, this review is devoted to the most promising NGs reported in the literature, which can be actually or potentially used in biomedical applications.

## Figures and Tables

**Table 1:** Stimuli-responsive nanogels and their main uses in biomedical applications.

Nanogels Based on	Synthesis Process	Drug	Stimuli-Responsiveness	Therapeutic Field	Reference
Methacrylate hyaluronic acid and di(ethylene glycol) diacrylate (MAHA-DEGDA)	Radical copolymerization	Doxorubicin	Physiological enzymes and pH	Chemotherapy	[142]
4-Methoxybenzoic acid-poly[(2-(pyridin-2-yldisulfanyl)-*co*-[poly(ethylene glycol)]-poly(*N*-isopropyl methacrylamide (MBA-PDA-PEG-PNIPAM)	Free radical polymerization	Silicon phthalocyanine photosensitizer, Pc4	pH, temperature and redox potential	Chemotherapy	[148]
Hyaluronic acid (HA)	Emulsion	Graphene and Doxorubicin conjugates	Light, temperature, and redox potential	Optical imaging and thermo-chemotherapy	[149]
Poly(ethylene glycol)-Hyaluronic acid with Ag-Au Nanoparticles (Ag-Au@PEG-HA)	Precipitation polymerization	Temozolomide	Temperature and light	Optical imaging and chemo-photo-thermal-therapy	[76]
Poly(*N*-isopropyl methacrylamide-methacrylic acid-quaternary ammonium alkyl halide with Fe_3_O_4_ nanoparticles P(NIPAM-MAA-DMAEMAQ) and poly(*N*-isopropyl methacrylamide)-methacrylic acid-hydroxyl ethyl methacrylate-quaternary ammonium alkyl halide with Fe_3_O_4_ nanoparticles P(NIPAAm-MAA-HEMA-DMAEMAQ)	Free radical polymerization	Doxorubicin and methotrexate	pH, temperature and magnetic field	Chemotherapy	[150]
4-Vinylphenylboronic acid-2-(dimethylamino)ethyl acrylate with Ag Nanoparticles (Ag@P(VPBA-DMAEA)	Emulsion polymerization	Insulin	Glucose and light	Diabetes treatment	[151]
Glycol chitosan-sodium alginate-poly(l-glutmate-*co*-*N*-3-l-glutamylphenylboronic acid) (GC/SA-PGGA)	Isotropic gelation method and electrostatic interactions	Insulin	Glucose	Diabetes treatment	[152]
Poly(*N*-isopropyl methacrylamide)-dextran-maleic acid-phenylboronic acid P(NIPAM–Dex–PBA)	Polymerization	Insulin	pH, temperature and glucose	Diabetes treatment	[153]
Hydroxypropylmethylcellulose/poly-(acrylic acid) (HPMC/PAA)	Surfactant free polymerization	Insulin	pH	Diabetes treatment	[154]
Poly(ethylene glycol)-poly(aspartic acid) (PEG-PAsp)	Self assembling and cross-linking	Insulin	pH	Diabetes treatment	[155]
Boronic acid-Fe_2_O_3_ Nanoparticles-poly(vinyl alcohol)-*b*-poly(*N*-vinylcaprolactam) PVOH-*b*-PNVCL	Micelle thermo-formation	Tamoxifen	pH, temperature, glucose and magnetic field	Thermo-chemotherapy and optical imaging	[156]
Dextran-(2-[methacryloyloxy]-ethyl) trimethylammonium chloride DEX-NGs	Inverse miniemulsion photopolymerization	siRNA	Physiological stimuli (natural pulmonary surfactant)	Pulmonary diseases	[157]
Dextran-Lysozyme (Ab-NG-DEX)	Enzymatic reaction	Antibody ICAM-1 and dexamethasone	Physiological stimuli (intercellular adhesion molecule-1)	Acute pulmonary inflammation	[158]
Poly(ethylene oxide)-poly(propylene oxide)-poly(ethylene oxide)-Polyvinyl alcohol with Fe_3_O_4_ nanoparticles (F-127-PVA)	Self-assembly	Ethosiximide	Magnetic field and temperature	Epilepsy	[159]
Poly(*N*-isopropyl methacrylamide and *N*-vinylpirrolidone (PNIPAM-VP)	Free radical polymerization	*N*-hexylcarbamoyl-5-Fluorouracil	Temperature	Brain tumors	[160]
Polysorbate 80-coated chitosan	Ionic gelation	Methotrexate	Surface modification	Brain tumors	[161,162]
Polyvinylpyrrolidone-poly(acrylic acid) (PVP/PAAc)	g radiation-induced polymerization	Dopamine	pH	Parkinson disease	[163]
Poly(ethylene glycol) and polyethylenimine PEG-PEI	Emulsification-solvent evaporation	Oligonucleotides	Surface functionalization	Brain diseases	[164]
Cholesterol-Polylysine	Emulsification-solvent evaporation	Nucleoside reverse transcriptase inhibitors	Surface functionalization	Human Immunodeficiency Virus (HIV)-associated encephalitis and neurodegeneration	[165]
Cholesterol-bearing pullulan (CHP)	Self-assembly	CHP nanogel membrane	Natural-based nanogels	Bone regeneration	[166]
Cholesterol-bearing pullulan (CHP)	Self-assembly	Prostaglandin E1	Natural-based nanogels	Wound healing	[167]
Cholesterol-bearing pullulan (CHP)	Self-assembly	W9-peptide	Natural-based nanogels	Bone regeneration	[168]
Cholesteryl group- and acryloyl group-bearing pullulan (CHPOA)	Self-assembly	Human bone morphogenetic protein 2 and recombinant human fibroblast growth factor 18	Natural-based nanogels	Bone regeneration	[169]
